# Treating emotional pains in parents of children with hearing impairment

**DOI:** 10.1097/MD.0000000000046076

**Published:** 2025-11-28

**Authors:** Anthonia Aneke, Chikodi Joy Anyanwu, Amanda Ugonna Ugwoezuonu, Moses Onyemaechi Ede, Beth Nnenne Oluka, Ifeanyichukwu D. Okoro, Nkechi T. Egenti, Obiagali Loretta Aniaku, Ifeanyichukwu B. Agbigwe, Joy Obiageli Oneli

**Affiliations:** a University of Nigeria, Nsukka, Enugu, Nigeria; b Delta State University, Abraka, Nigeria; c University of Johannesburg, Johannesburg, South Africa; d Ebonyi State University, Abakaliki, Nigeria; e Pastoral Ministry Grace Training International Bible Institute, Duluth, Gambia.

**Keywords:** emotional pains, family health model, hearing impairment, management, parents of children, rational emotive behavior therapy

## Abstract

**Background/objective:**

Sustainable well-being is an important global health issue. Unfortunately, the psychosocial well-being of children and adult populations with disabilities is being threatened in several ways in Nigeria. Consequently, they experience an increased risk of mental health conditions, but there are limited quantitative studies and few available mental health support for them and their caregivers. This study focused on the effect of the family health model in managing emotional pain in parents of children with hearing impairment in Nigeria.

**Methods:**

This was implemented using a group randomized controlled design, and a sequence allocation method was used to assign 58 participants to the intervention group and 59 parents to the comparison group. Two measures were used to measure the participants’ emotional pains and irrational beliefs at the pretest, post-test, and follow-up test. Participants in the treatment group were exposed to a 12-session intervention.

**Results:**

The baseline test revealed that there was no significant difference in the mean scores of the participants in the treatment group and those in the waitlisted control group. The post-treatment test revealed a significant positive change in treatment group participants. The third assessment revealed that the significant improvement in participants in the treatment lasted over time. It was also shown that gender does not influence the outcome of treatment.

**Conclusion:**

The Rational Emotive Family Health model is an effective strategy in reducing and treating emotional pain in parents of children with hearing impairment. Based on the findings, recommendations were established. That awareness creation to inform people, especially young and older adults the importance of mood monitoring and regular medical assessments.

## 1. Introduction

Hearing impairment (HI) is a disability that affects the individual sense of perceiving sound, and perfect functioning of the total personality without minding the period of onset.^[1]^ According to the Individuals with Disabilities Education Act HI whether permanent or fluctuating is a disability that adversely affects a child’s educational performance. HI could be classified as deafness or hearing loss.^[2]^ Deafness occurs when the hearing loss is above 90 decibels and is regarded as HI when the decibel is below 90 decibels.

HI decreases the rate of hearing and communication among the population affected within their surroundings.^[3]^ Researchers have indicated that HI influences communication, cognitive functioning, increased psychopathological symptoms in children,^[4,5]^ academic performance and psychosocial functioning,^[6]^ and social development like positive relationships with others.^[7]^ Prior studies have equally documented that HI adversely affects language acquisition, quality of communication, social skills development, capabilities, self-confidence, and cognitive and emotional skills.^[2]^ This indicates that children with HI could likely find it difficult to express feelings and make coherent communication specifically at home, in a public gathering, and in the midst of peers. Thus, this adversely tends to inflict emotional pains among the parents of children with HI.

It is notably common among parents to experience trauma when a child is diagnosed with a disability like HI.^[8]^ In the researcher’s close contact and interactions, with dismay, we observed common incessant suicide ideation, high rate and increased blood pressure, wretchedness, drug abuse, quarreling, marital divorce, and negative self-denials among parents with hearing disabled children. These are supported by the findings of Anclair et al^[8]^ and Anclair et al^[9]^ that parents experience fear, burden, and shock due to health conditions and the future of their child with a disability. Other studies acknowledged deteriorating quality of life,^[10]^ stress-related disarrays,^[11]^ and emotional pains.^[12]^ These experiences appear to be common among parents with a hearing-impaired population compared to parents of normally developing children.^[11]^

More so, scholars in related studies validate the challenges and emotional pains experienced by parents of disadvantaged children HI inclusive,^[13]^ type 1 diabetes,^[14]^ chronic pain,^[11]^ and highly isolation type of life.^[15]^ The findings of the qualitative analysis of parents with hearing-impaired children diagnosed showed that parents experienced such psychological crises like guilt, anger, and pain,^[16]^ and frustration.^[17]^ Equally, the result of the previous studies indicated that parenting populations with HI increased context-specific stress like emotional pains when compared to parents of normal hearing children.^[16,18]^

Pressure is another aspect of tensions and emotional pains parents experience in caring for their disabled child.^[19]^ Pain is an unpleasant sensory and emotional experience associated with actual or potential tissue damage.^[20]^ Pain could be acute or chronic.^[21]^ Chronic pain occurred from damage to the central nervous system and is described as stabbing, shooting, or burning.^[22]^ Schechter et al^[22]^ describe acute pain as rising from a short episode of tissue injury or inflammation and lasting a short period of time. Thus, parenting children with varied disabilities like autism spectrum disorder, visual impairment, intellectual disability, and HI population could induce emotional distress leading to psychological crises that affect health like heart failure, increase blood pressure, and trigger asthma attacks.^[23–25]^ This could be seen in the constant expenses that the family will be incurring to keep life moving. Thus, literature has shown that parents of children with other disabilities experience parental pains but parenting a child with HI is higher as it is negatively affecting the overall wellness of the family.^[26]^

Globally, studies have been conducted on the prevalence of emotional pains among parents of the population with disabilities. The findings of the study conducted in Pakistan indicated that 74% of parents noted that the existence of HI in the family is a source of economic tension.^[27]^ The author reported further that 66% acknowledge societal challenges, 84% indicated an increased loss of appetite and fears while 44% noted an increased worry, acute headaches, and severe emotional pains.^[27]^ In a study conducted in Malawi, over 40% of emotional pain among parents of an individual with an intellectual disability was recorded.^[28]^ Studies in Kenya also recorded above 70% prevalence of depression among the parents of children with varied disabilities.^[29]^ In Ireland over 90% prevalence of emotional distress among the population of parents with disabilities was recorded compared to parents of typically developing children.^[30]^ Other studies recorded 30% to 40% of symptoms of psychological distress among parents parenting disabled children.^[31]^

In Nigeria, over 40% of parents were reported to be psycho-emotionally distressed for parenting patients with sickle cell disease anemia.^[32]^ Yusuf and Nuhu^[33]^ reported 70% and above emotional distress among parents of individuals with a disability. Other studies reported an over 70% increase in emotional depression among parents of human immunodeficiency virus positive children.^[34]^ Above 53% of emotional distress was also reported among Nigerian couples.^[35,36]^ Thus, this indicates that emotional distress is in a high increase among parents parenting populations with HI and other related disabilities.

Parents perceive emotional distress differently according to gender. Azeem et al^[37]^ reported above 80% depression among mothers and 77% among fathers in a study to assess the level of depression and both anxiety and depression among parents of children with intellectual disabilities in Pakistan. Tan and Rey^[38]^ indicated high emotional distress among mothers than fathers of children with disability. Emotional distress, anxiety, and depression are common among mothers to fathers of an individual with varied disabilities.^[39]^ Recent studies have noted the existing comparable level of distress among parents parenting individuals with varied disabilities including HI.^[40]^

Irrational belief could induce emotional distress. Parents who hold irrational thoughts regarding their situations due to the existence of a disability in the family could be pronto to increase emotional crises like heart attack and loss of appetite. Given the steady increase of emotional distress among the parents of the population with HI and the psychological consequences on parental wellness, it becomes consequential to validate if rational emotive behavioral therapy (REBT) could be useful in decreasing the emotional distress of parents parenting children with HI. Notably, studies have suggested that REBT be employed to alter the irrational thinking of the parents of children with varied disabilities to achieve optimum life goals and healthiness.^[25,41]^ Given this claim, therefore, we argued that there exists a lack of studies designed to help parents of children with HI in Enugu state, manage and decrease the rate of emotional distress often experience, and improve their well-being. We hypothesized that the level of emotional distress would be decreased following the applications of family health model of REBT amidst the therapeutic group when compared to control group. As an extension of REBT by Ellis^[42]^ which holds that an individual’s irrational belief, affects their thought and behavior. Thus, the current study adopted a family health model of REBT to help in altering irrational thoughts and existing negative beliefs among parents with hearing-impaired populations. Family health model of REBT coined by Ede et al^[25]^ was initially called rational emotive family health therapy (REFHT) and it is called family health model in this study since it is an extension of REBT which seeks to address family health issues as associated with rationality and irrationality.

### 1.1. Family health model of REBT

In line with REBT principles, the rational emotive family health model (REFHM) was coined.^[25]^ REFHM is an evidence-based intervention program targeted at decreasing irrational thoughts and erroneous beliefs that generate an unhealthy interpretation of realities among parents parenting populations with HI. In other words, parents that hold erroneous or irrational belief regarding situations will not achieve reality.^[43]^ Also, family interpretations and beliefs about challenges in the family could determine the level of emotional distress. Thus, if the challenging situation is erroneously interpreted, the family could be vulnerable to high emotional distress. Hence, we contend that unhealthy interpretations occur once a gap exists between human rationality and the actual existence of the situation.

Even though families are increasingly being acknowledged as an important part of health care, no overall family intervention framework has become widely accepted as a means of treating emotional pains in the family context. Among the frameworks, for instance, the health-promoting family concept, describes how families promote members’ health, including children. As experts in designing interventions and researchers are yet to accept a specific family-emotion-health-focus therapy, the present study was poised to adapt the family health model of REBT.

Erroneous interpretations of events arise when parents parenting populations with disabilities (hearing impaired) accept within themselves that they cannot cope amide of the situations and challenges related to their child’s disability, again when family lives to isolate and wallows in the low esteemed type of life when in their thought they believed that they cannot make life worth living. The result could predispose the individual family to trauma, depression, frustrations, and emotional distress.^[25,43,44]^

Prior studies have indicated that erroneous justification and belief regarding coping up with the challenges of parenting hearing-impaired populations and self-defeating have a significant impact on the parental relationship.^[44,45]^ Equally, substantial evidence has shown that behaviors worth unacceptable by individuals could be attributed to erroneous self-beliefs.^[25,45,46]^ Also, erroneous self-beliefs regarding parenting hearing-impaired children could perhaps, affect the wellness of the entire family both physical and psychological growth, hence trauma, and emotional distress experienced.^[46]^ Similarly, psychological issues regarding coping with parenting a child with disability and possibly intervention could as well induce erroneous beliefs and irrational behavior.^[25,46,47]^ Researchers have indicated that parental irrational beliefs could be classified according to parental beliefs.^[46,48]^ Examples of such parental irrational beliefs are demandingness, awfulizing, low frustration tolerance, and global evaluation of human worth.^[49,50]^ Demandingness occurs when parents become so rigid and strict regarding the child’s wellness, ought or should be belief. Awfulizing has to do with a situation where parents belief that a certain situation is unbearable or too difficult to resolve. Low frustration tolerance is a belief that certain situations could be unbearable. Global evaluation of human worth is a thought that family respect, worth, and dignity are depending on the achievements skills of an individual.^[48]^ Parents with these characteristics’ beliefs could be described as having irrational and unhealthy beliefs and feelings, hence distortions of reality are experienced.^[25]^ The authors further stated that parents with these belief systems may be described as having a negative and unrealistic beliefs and feelings and by so doing, actualizing the reality of the situation would be distorted.^[25]^

Findings from previous studies have shown that parents of children with disability (hearing impaired) could be pronto increased risk of emotional distress, unhealthy emotions, and psychological and behavioral responses regarded as poor physical and emotional wellness.^[51,52]^ Thus, these psychological response affects both the physical and emotional wellness of the entire family specifically the parents. From the REFHT viewpoint, parents that chose to isolate, indulge in excessive alcohol, abuse drug, exhibits worry, and nags as a result of people thinking regarding disability (hearing loss) amidst the family could be vulnerable to emotional distress.^[52,53]^ A lot of evidence abounds indicating the high benefits of rational emotive behavior approaches in sustaining healthy emotional and behavioral functions among parents parenting individuals with varied disabilities and HI inclusive.^[53,54]^ Rational emotive family health approach has been conducted using other populations like parenting stress in families of children with autism spectrum disorders.^[25]^ REBT has also been used among Romanian foster parents with psycho-emotional distress.^[55]^ Joyce^[45]^ equally developed a rational emotive parent education program to reduce emotional stress among 48 parents. In line with the literature reviewed and the researcher’s observation, we observed that REFHT is targeted to assist parents to identify, disputing, and decreasing parental erroneous thought and beliefs associated with parenting populations with disabilities and educates parents on how to adjust.^[25,45]^ Thus, within the context of this study, it is likely that psycho-emotional crises like self-worry, economic crises, increased loss of appetite, and sleepless nights could generate pressure, trauma, and acute emotional distress among parents parenting vulnerable children. It, therefore, becomes imperative to adopt the family health model of REBT to assist parents of children with HI to manage and improve on emotional distress that has been a barrier to overall family development.

## 2. Methods

### 2.1. Study design

The design of this research is a pretest-posttest group design with a follow-up. A pretest-posttest group design with follow-up is frequently used to evaluate the efficacy of programs or treatments. It entails assessing a dependent variable before and after an intervention, then again at a later time point to assess the intervention’s long-term effects.

### 2.2. Participants and procedure

Before the commencement of the intervention, the research assistants visited 2 special schools to inform the management and sort for the phone numbers, WhatsApp numbers, and email addresses of the parents of children with HI within the study center. Invitation to participate in the program was widely publicized so as to accommodate every parent whose children are already dropped out of school, hence the particulars of such parents were not in school. This study employed every means within our reach, viz use of fliers, being bored, announcing the participations in places of worship, and using local means of communication.

The participants include parents whose children were diagnosed with HI. After disclosing the objectives of the study, the participants were notified that participation was voluntary and that they could withdraw from the study voluntarily without any consequences. The recruitment lasted between January and March.

**Figure 1.**
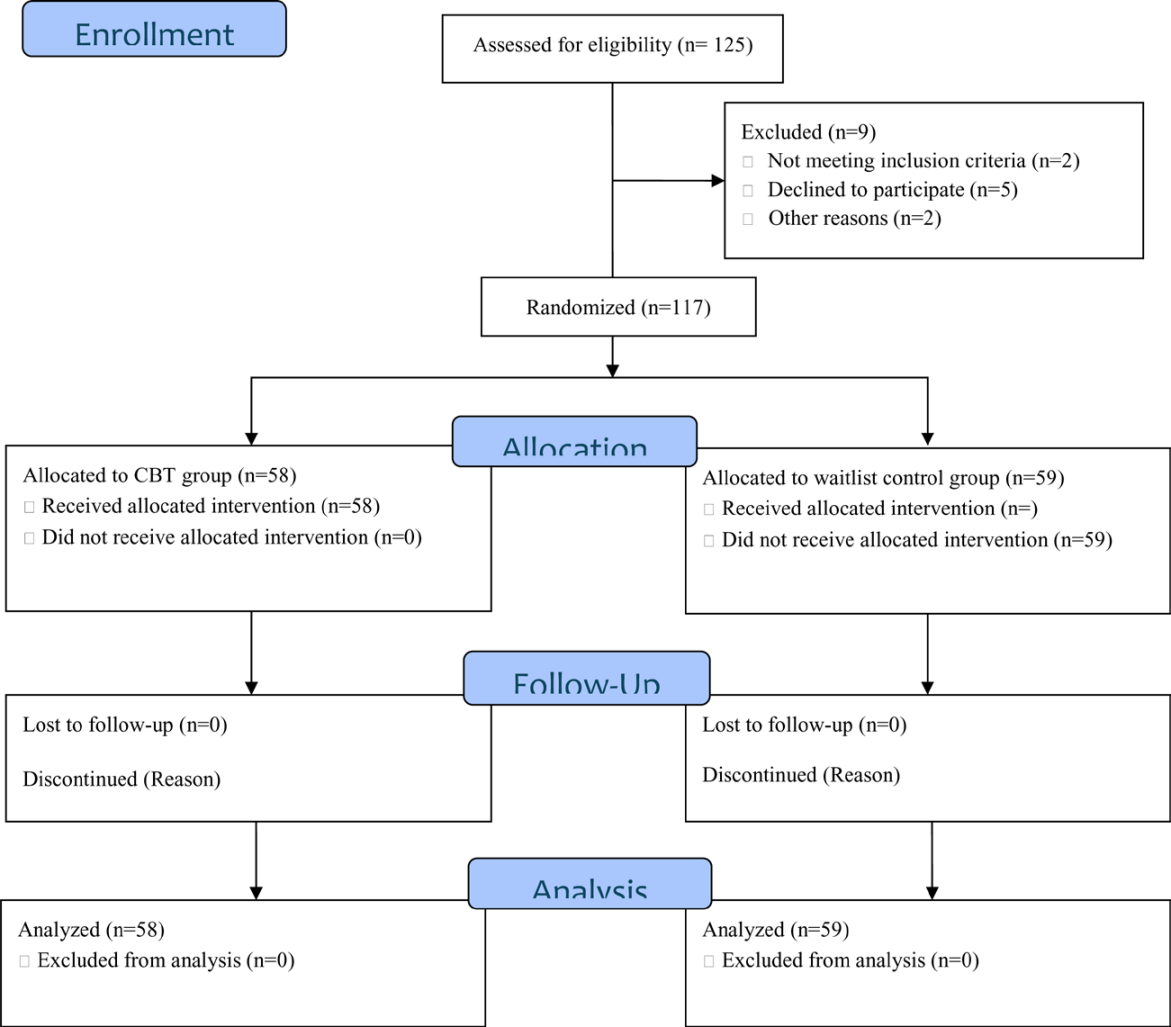
CONSORT participant eligibility flowchart. CBT = cognitive behavioral therapy, CONSORT = Consolidated Standards of Reporting Trials.

About 126 parents were in attendance but 117 parents measured to our stated criteria. See Figure 1 for more details on randomization. Parents whose children were diagnosed in line with the Diagnostic and Statistical Manual of Mental Disorders IV criteria were included in this study. Other inclusion criteria considered during screening are the parents must be 18 years and above, must have a functional mobile phone, sign the participant’s consent form, and not have received any counseling/mental treatment from a cognitive therapist in the last 7 years. Those who lost short of the stated criteria were excluded from the study. Profile of Mood States (POMS) and Perceived Emotional Distress Inventory (PEDI) were the instrument used to obtain baseline data for this study of emotional distress of parents parenting populations with HI.

The 117 participants (parents of children with HI diagnosed) formed the sample size. The power of the sample size was calculated using GPower 3.1 software.^[56]^ The power analysis gave a power of 0.94 which showed that the sample size was adequate. Refer Table 1.

**Table 1:** Sociodemographic information of the participants based on groups.

	REFHI group, n (%)	Control group, n (%)	Statistic (χ^2^)	Sig.
Gender				
Male	27 (46.6)	24 (41.4)	0.315	.575
Female	31 (53.4)	35 (58.6)		
Age (yr)				
20–30	3 (5.2)	7 (70.0)	4.153	.245
31–40	9 (15.5)	13 (22.4)		
41–50	19 (32.8)	20 (34.5)		
51 and above	27 (46.6)	18 (31.0)		
Family size				
Small size	16 (27.6)	9 (15.5)	2.508	.285
Moderate size	21 (36.2)	24 (41.4)		
Large size	21 (36.2)	25 (43.1)		
Marital status				
Single parent	5 (8.6)	8 (13.8)	0.862	.650
Married	28 (48.3)	25 (43.1)		
Divorce	25 (43.1)	25 (43.1)		
Socioeconomic status				
Low SES	5 (8.6)	4 (6.9)	0.376	.829
Moderate SES	14 (24.1)	12 (20.7)		
High SES	39 (67.2)	42 (72.4)		

REFHI = rational emotive family health intervention, SES = socioeconomic status, sig = associated probability.

Paper cards were packed inside a brown envelope labeled “C” (Choosing for the intervention group) and “N” (Not choosing for the intervention group) to randomly assign participants to 2 different groups. Using “C” and “N” to represent the intervention arms aimed to enhance concealment and control potential selection bias during the allocation process. This method resulted in 58 parents being assigned to the treatment group and 59 parents to the no-treatment group. Participants in the no-treatment group did not receive any form of intervention. See Figure 1 for more details.

The participants in REFHM group were exposed to a 12-session treatment program aimed at addressing the psycho-emotional related problems challenging parents parenting population with HI. In sessions 1 to 3, the therapist focused on general introduction, establishing rapport, principles, and guiding rules among the participants, the meaning of emotional distress, and the possible causes and symptoms. Sessions 4 and 5 reflected on the relationship between emotional pains and HI and how parents’ perceptions regarding HI could induce emotional pains. Session 6 addressed how poor interpretations of the reality facing a family member lead to distorted family development, family relationships, dynamic interactions between family members, and family health practices. Session 7 focus on familiarizing the participants with the REFHM, purpose, and approach. Sessions 8 and 9 focus on assisting the participant to identify and clarify irrational thoughts and unhealthy emotions and beliefs generating emotional distress as a result of parenting HI children. Sessions 10 and 11 deal with helping the participant discover how to recognize normal beliefs and rational self-statement with automatic and wholistic disputation of irrational thought which has been identified as the cause of emotional distress and replacing those irrational/unhealthy emotions with rational and healthy ones so as to cope up with the challenges of emotional distress and enjoy psychological and emotional healthiness. Finally, session 12 focused on the revision of the previous exercise and termination. Similarly, the participants at REFHT program were given writing materials with a summary of lecture notes presented during each session for home practice and assignments.

The psycho-intervention program was delivered by 2 family health therapists and 2 counseling psychologists. All the therapists employed for this study have PhD in counseling psychology and have practiced rational-emotive and cognitive behavioral therapy (CBT) for over 10 years. The intervention was equally delivered using English language and Igbo Language. The researchers equally employed the services of a signed language interpreter who did practical demonstrations of sign language exercises with the participants. Treatment delivery assessors were recruited by the researchers to monitor day-to-day activities of the participants and therapists. This was to ensure that manual was implemented as expected.

All the participants completed the 12-session program, and as such, there was no record of any cases of dropout. To achieve maximum compliance from the selected participants, we made a provision of 4 mini shuttle buses for easy transportation, as a means of support and to encourage strict adherence to attendance. Equally, there was the provision of food, snacks, soft drink, and financial assistance of 5 USD per attendance. At the end of the therapeutic program, the participants in treatment and no-contact control groups were evaluated at Time 2. The researchers with the help of research assistants, later met the participants in a follow-up meeting, which lasted for 1 month after 6 months. The follow-up meeting occurred once per week, and it focused on the assessment of participants for the third point (Time 3) and the termination of the therapy. The data analysts were blinded to enhance concealment and control possible bias during the data analyses.

### 2.3. Measures

POMS is an instrument developed by McNair et al.^[57]^ The instrument is a 65-item that assesses psychological distress experienced by an individual. The 65 items are subdivided into 6 subscales like anger, depression, fatigue, tension, vigor, and confusion-bewilderment. Specifically, the anger subscale assessed hostility and irritability, depression subscale assessed feelings of isolation, guilt, futility, and sadness, fatigue subscale assessed feelings of weariness, tension subscale assessed the subjective state and somatic experience of anxiety, vigor (positive mood state) subscale assessed wellbeing, enthusiasm, liveliness, energy, and optimism, and confusion-bewilderment (negative mood states) subscale assessed efficiency and clarity of thinking. POMS is rated on 5-point Likert scale format ranging from 0 = not at all, 1 = a little, 2 = moderately, 3 = quite a bit, and 4 = extremely. The POMS scale has been acknowledged to be valid and reliable when used in other domains (e.g., sports). The short forms were found to be as reliable as the long form when measuring individual issues. A past research established the reliability (Cronbach alpha = 0.81 and 0.90 as internal consistency) of the scale in this study.^[58]^ The present researchers trial-tested the measure to understand the cultural relevance in Nigeria. The internal consistency after testing its validity and reliability in Nigeria was α = 0.79.

PEDI was developed by Moscoso et al.^[59]^ PEDI is a 15-item self-report screening scale designed to assess the presence and severity of anxiety, anger, depression, and hopelessness among patient populations. PEDI is measured on a 4-point Likert scale format from 0 = not at all, 1 = sometimes, 2 = often, and 3 = very much so. The PEDI total score ranges from 0 to 45 points, in other words, the higher scores correspond to higher levels of emotional distress perceived. We establish the reliability (Cronbach alpha = 0.86 as internal consistency) of the scale in this study. The validity and reliability of emotional distress have been established across cultures and countries like United States,^[60]^ Canada,^[61]^ and Nigeria.^[62]^ The measure was trial-tested by the current researchers in order to determine its cultural relevance in Nigeria. After being tested for validity and reliability in Nigeria, the internal consistency was α = 0.77.

### 2.4. Intervention

REFHM was coined by Ede et al.^[25]^ The focus is to adapt the fundamental principles of REBT to redress the psycho-emotional distress confronting parents parenting children with HI. We adapted REFHT manual developed^[25]^ for this study. The manual is a psycho-education and therapeutic program designed to help parents to alter irrational thoughts related to parenting the hearing impaired population and associated emotional distress. The manual consisted of 12 weeks of 12 sessions that lasted for 50 minutes at each meeting. A lot of techniques are contained in the manual like meditation,^[63,64]^ behavioral exercises,^[65]^ relaxation techniques,^[66]^ and biofeedback^[67]^ including other techniques REBT therapist have used.

### 2.5. Data analysis

The data from the study were subjected to statistical analysis using SPSS version 28. Specifically, the multivariate method of data analysis ascertained the effectiveness of REFHT in managing emotional pains and mood disturbance among parents of children with HI. The effect size of the intervention was reported using partial eta square.

## 3. Results

As in Table 1, there is no significant difference between participants in the treatment group and those in the comparison group with regard to gender (χ^2^ = 0.315, *P* = .575), age (χ^2^ = 4.153, *P* = .245), family size (χ^2^ = 2.508, *P* = .285), marital status (χ^2^ = 0.862, *P* = .650), and socioeconomic status (χ^2^ = 0.376, *P* = .829).

Table 2 shows the descriptive analysis of the responses of the participants per group. The total mean (standard deviation) of emotional state scores at the pretest, posttest, and follow-up test for REFHT group was 226.76 (4.21), 204.40 (6.29), and 200.48 (8.01) as measured by POMS, respectively. As measured by PEDI, the total mean (standard deviation) of emotional state scores at the pretest, posttest, and follow-up test for REFHT group was 40.92 (1.62), 38.30 (1.81), and 38.09 (1.76). The results imply that participants’ depression mean and standard deviation scores positively changed from the first assessment to the last assessment. On the other hand, the total mean (standard deviation) of control group participants’ depression scores at the pretest, posttest, and follow-up were 228.58 (3.85), 229.63 (3.73), and 228.65 (3.91) as measured by POMS, respectively. Also, the total mean (standard deviation) of emotional state scores at the pretest, posttest, and follow-up test for control group was 41.05 (1.34), 39.27 (1.51), and 39.49 (1.75) as measured by PEDI indicating that there was no change rather a steady increase in the mean depression value scores observed among participants in the no-contact control group over time.

**Table 2:** Descriptive statistical analysis of participants responses across groups and time.

Time of measurement	Group	Gender	POMS, Mean (SD)	PEDI, Mean (SD	N
Pretest	Treatment group	Male	225.90 (3.51)	40.93 (1.37)	27
		Female	227.50 (4.66)	40.92 (1.84)	31
		Total	226.76 (4.21)	40.92 (1.62)	58
	Control group	Male	227.64 (3.34)	41.09 (1.36)	24
		Female	229.25 (4.08)	41.02 (1.34)	34
		Total	228.58 (3.85)	41.05 (1.34)	58
Posttest	Treatment group	Male	204.91 (6.28)	38.61 (1.55)	27
		Female	203.96 (6.36)	38.04 (2.00)	31
		Total	204.40 (6.29)	38.30 (1.81)	58
	Control group	Male	228.67 (3.28)	39.41 (1.38)	24
		Female	230.31 (3.93)	39.17 (1.60)	34
		Total	229.63 (3.73)	39.27 (1.51)	58
Follow-up	Treatment group	Male	199.01 (8.68)	37.97 (1.48)	27
		Female	201.76 (7.29)	38.19 (1.99)	31
		Total	200.48 (8.01)	38.09 (1.76)	58
	Control group	Male	227.96 (3.64)	39.49 (1.81)	24
		Female	229.14 (4.07)	39.49 (1.74)	34
		Total	228.65 (3.91)	39.49 (1.75)	58

PEDI = Perceived Emotional Distress Inventory, POMS = Profile of Mood States.

Table 3 shows the multivariate analysis of the effect of the intervention on emotional pains. The multivariate result shows that emotional pains responses of the participants in REFHT and control groups at the pretest stage have no significant difference as measured by the POMS, *F*(1, 115) = 5.456, *P* = .21, ∆*R*^2^ = 0.063, respectively. The participants were assessed a second time after the REFHT treatment and results show a significant positive change in treatment group participants as measured by POMS, *F*(1, 115) = 668.881, *P* < .001, $\eta_{p}^{2}=0.857$, ∆*R*^2^ = 0.857, respectively. The third assessment revealed that the significant improvement in participants in the treatment was lasted over time as measured by POMS, *F*(1, 115) = 575.003, *P* < .001, $\eta_{p}^{2}=0.837$, ∆*R*^2^ = 0.836, respectively. The effect size values of 0.703 and 0.769 at posttreatment and follow-up assessment represent that 80.6% and 80.4% significant sustained changes were attributed to REFHT treatment.

**Table 3:** Multivariate analysis of the effect of REFHT on emotional pains as measured by POMS.

Source	Dependent variable	Type III sum of squares	df	Mean square	*F*	Sig.	Partial eta squared
Corrected model	POMSPretest	170.550*	3	56.850	3.581	.016	0.088
	POMSPosttest	18,513.527†	3	6171.176	230.705	<.001	0.861
	POMSFollowUp	23,148.526‡	3	7716.175	196.236	<.001	0.840
Intercept	POMSPretest	5,903,061.872	1	5,903,061.872	371,881.865	<.001	1.000
	POMSPosttest	5,365,509.920	1	5,365,509.920	200,586.075	<.001	0.999
	POMSFollowUp	5,242,688.187	1	5,242,688.187	133,330.695	<.001	0.999
Group	POMSPretest	86.605	1	86.605	5.456	.021	0.046
	POMSPosttest	17,892.006	1	17,892.006	668.881	<.001	0.857
	POMSFollowUp	22,609.642	1	22,609.642	575.003	<.001	0.837
Gender	POMSPretest	73.684	1	73.684	4.642	.033	0.040
	POMSPosttest	3.411	1	3.411	0.128	.722	0.001
	POMSFollowUp	109.391	1	109.391	2.782	.098	0.024
Group × Gender	POMSPretest	0.002	1	0.002	0.000	.991	0.000
	POMSPosttest	47.719	1	47.719	1.784	.184	0.016
	POMSFollowUp	17.667	1	17.667	0.449	.504	0.004
Error	POMSPretest	1777.831	112	15.873			
	POMSPosttest	2995.906	112	26.749			
	POMSFollowUp	4403.945	112	39.321			
Total	POMSPretest	6,014,678.755	116				
	POMSPosttest	5,484,768.242	116				
	POMSFollowUp	5,367,949.553	116				
Corrected total	POMSPretest	1948.381	115				
	POMSPosttest	21,509.433	115				
	POMSFollowUp	27,552.471	115				

POMS = Profile of Mood States, REFHT = rational emotive family health therapy.

* *R*^2^ = 0.088 (adjusted *R*^2^ = 0.063).

† *R*^2^ = 0.861 (adjusted *R*^2^ = 0.857).

‡ *R*^2^ = 0.840 (adjusted *R*^2^ = 0.836).

The result shows that gender does not influence the outcome of REFHT treatment, *F*(1, 115) = 0.128, *P* = .722, $\eta_{p}^{2}=0.001$. The significant interaction effect of groups and gender during treatment does not exist with regard to participants’ emotional pains scores (*F*(1, 115) = 1.784, *P* = .184, $\eta_{p}^{2}=0.16$).

Table 4 shows the multivariate analysis of the effect of the intervention on emotional pains. The multivariate result shows that emotional pains responses of the participants in REFHT and control groups at the pretest stage have no significant difference as measured by the PEDI, *F*(1, 115) = 0.208, *P* = .002, ∆*R*^2^ = 0.025, respectively. The participants were assessed at second time after the REFHT treatment and results show a significant positive change in treatment group participants as measured by PEDI, *F*(1, 115) = 9.545, *P* = .003, $\eta_{p}^{2}=0.079$, ∆*R*^2^ = 0.070, respectively. The third assessment revealed that the significant improvement in participants in the treatment was lasted over time as measured by PEDI, *F*(1, 115) = 18.195, *P* < .001, $\eta_{p}^{2}=0.140$, ∆*R*^2^ = 0.119, respectively. The effect size values of 0.703 and 0.769 at posttreatment and follow-up assessment represent that 70.0% and 14.0% significant sustained changes were attributed to REFHT treatment.

**Table 4:** Multivariate analysis of the effect of REFHT on emotional pains as measured by PEDI.

Source	Dependent variable	Type III sum of squares	df	Mean square	*F*	Sig.	Partial eta squared
Corrected model	PEDIPretest	0.512*	3	0.171	0.076	.973	0.002
	PEDIPosttest	32.446†	3	10.815	3.887	.011	0.094
	PEDIFollowUp	57.935‡	3	19.312	6.183	<.001	0.142
Intercept	PEDIPretest	191,490.953	1	191,490.953	84,975.809	<.001	0.999
	PEDIPosttest	171,643.496	1	171,643.496	61,686.518	<.001	0.998
	PEDIFollowUp	171,466.715	1	171,466.715	54,894.528	<.001	0.998
Group	PEDIPretest	0.468	1	0.468	0.208	.650	0.002
	PEDIPosttest	26.559	1	26.559	9.545	.003	0.079
	PEDIFollowUp	56.834	1	56.834	18.195	<.001	0.140
Gender	PEDIPretest	0.057	1	0.057	0.025	.874	0.000
	PEDIPosttest	4.656	1	4.656	1.673	.198	0.015
	PEDIFollowUp	0.357	1	0.357	0.114	.736	0.001
Group × Gender	PEDIPretest	0.021	1	0.021	0.009	.924	0.000
	PEDIPosttest	0.771	1	0.771	0.277	.600	0.002
	PEDIFollowUp	0.354	1	0.354	0.113	.737	0.001
Error	PEDIPretest	252.389	112	2.253			
	PEDIPosttest	311.641	112	2.783			
	PEDIFollowUp	349.839	112	3.124			
Total	PEDIPretest	195,102.590	116				
	PEDIPosttest	174,845.818	116				
	PEDIFollowUp	174,945.707	116				
Corrected total	PEDIPretest	252.901	115				
	PEDIPosttest	344.087	115				
	PEDIFollowUp	407.774	115				

PEDI = Perceived Emotional Distress Inventory, REFHT = rational emotive family health therapy.

* *R*^2^ = 0.002 (adjusted *R*^2^ = −0.025).

† *R*^2^ = 0.094 (adjusted *R*^2^ = 0.070).

‡ *R*^2^ = 0.142 (adjusted *R*^2^ = 0.119).

The result shows that gender does not influence the outcome of REFHT treatment, *F*(1, 115) = 1.673, *P* = .198, $\eta_{p}^{2}=0.015$. The significant interaction effect of groups and gender during treatment does not exist with regard to participants’ emotional pains scores (*F*(1, 115) = 0.277, *P* = .600, $\eta_{p}^{2}=0.02$).

## 4. Discussion

This research investigated the efficacy of the family health model of REBT in managing the emotional pains among parents parenting population with HI in Enugu state, Nigeria. The result showed that the family health model of REBT had a significant effect in cushioning emotional distress among parents parenting children with HI exposed to cognitive intervention compared to those in the waitlisted control group. Noteworthy, the impact of the intervention of emotional distress among parents with hearing-impaired children was maintained at follow-up. Equally, this improved level of emotional pain among parents of individuals with disabilities was attributed to the family health model of REBT. The result of this current study supported Ciff et al^[55]^ that REBT is effective in decreasing emotional distress. Ede et al^[25]^ noted that REFHT is effective in decreasing parental distress among parents of children with varied disability. The effectiveness of REBT was equally observed among Nigerian married couples.^[36]^ Psychological problems like trauma, stress, and illness could be associated with emotional distress.^[21,22,68]^ This indicates that REFHM like ours could be very useful in decreasing psychological/nonclinical and emotional problems among parents parenting populations with HI, specifically, if the problem is related to erroneous thinking and unhealthy behavior.

The findings of this study also aligned with the previous literature that validates the efficacy of REBT in mitigating emotional distress among Romanian foster parents.^[45]^ Also, the study conducted by Ede et al^[25]^ among parents parenting children with autism spectrum disorder indicated that rational emotive family health intervention is an evidence-based therapy. As an evidence-based approach, Gaviţa et al^[69]^ noted that CBT is effective in decreasing anxiety disorders, like anger and aggressiveness in treating persons with disability. By indications, emotional distress stems from an irrational belief. If parents parenting populations with HI continue to hold irrational thoughts regarding people’s perception of disability, stigmatization, uphold low self-esteem, attribute poor income level status to disability in the family, the future reality may be too difficult to achieve, hence, they (parents) are pronto psychological crisis like trauma, loss of appetite, and sexual urge, as well as emotional distress.^[24]^ Thus, a nonclinical intervention like ours is required.

Similarly, our findings support the study that revealed the effectiveness of REBT as an intervention approach among parents with emotional behavoiur.^[70]^ Mangayarkarasi and Sellakumar^[71]^ indicated that the application of REBT was very effective in decreasing the high level of depression among women affected with human immunodeficiency virus. Like our posttest evaluation result, the cancer patients exposed to psychological treatment recorded less pain compared to those in the control group.^[72]^ The authors further validated the effectiveness of psychological intervention for parents in improving parenting behaviors. Like other populations, REFHT has been shown to be promising among parents of the population with Down syndrome,^[54]^ and parents of exceptional children.^[46]^ Invariably, the findings of this study have equally validated the promising impact of REBT on parental attitudes after exposing parents to CBT sessions.^[25,73,74]^

This study recorded some strengths that added credence to it. The sample size was very much adequate for this study. The result obtained during the follow-up assessment justifies the effectiveness of REFHM in decreasing the cause-effect of emotional pains among parents parenting children with HI. The study also recorded 99.7% commitment of both the participants and the therapist. Hence, during the votes of thanks their spokesperson requested that REBT program be organized from time to time. The presence and activities of a sign language interpreter also added juice to the program. Another unique value added in this study was recognizing and teaching the participants the roles of irrationality on emotions of family members and how it affects family social context. Lastly, REFHM like ours was the first to be conducted among parents parenting children with HI in Enugu state and also the first to have a definitive diagnosis of HI disability (through the use of the Diagnostic and Statistical Manual of Mental Disorders IV) criteria. Meanwhile, the inflexibility of the criteria stated for the study could not allow many parents to participate in this study, and as such many dropped out. Also, the degree of HI among children was not measured as such, and the severity of the condition may have a significant effect on the parental emotions and psychological hence emotional distress experienced. We equally noted that some parent was subjective during the collection of data at time 1 specifically, parents from a rural setting.

Despite the contributions to knowledge that this study has made, there are limitations, including the lack of assessment of children’s HI severity, the potential influence of incentives (such as transportation and stipends), and the absence of long-term follow-up beyond 6 months. We encourage future researchers to incorporate and address these methodological gaps and flaws in subsequent studies.

### 4.1. Implications and future directions

The findings of this study have practical implications for practitioners. Given the findings, there is a need for the creation of rational emotive family health institutes or centers that would provide psycho-emotional interventions to support parents of children with HI to decrease their emotional distress. To facilitate this, REBT and childcare experts should collaborate in providing social and counseling services. The implication also extends to the need to provide education targeted at decreasing parental emotional distress among parents of children with varied disabilities in Enugu state by practitioners. They could engage in measuring the functional severity of the child’s disability as well as the specific diagnostic conditions that are more linked to parental participation and support. Reporting the health impact to government and nongovernmental bodies could attract other social supports to assist parents who may not be able to provide necessary support to children with HI. Practitioners could also advocate for families of children with HI.

REBT and family counselors are called to provide informational counseling by providing knowledge about hearing loss and its management, with relatively little information regarding feelings and emotions. The children with HI as well as their parents will receive counseling and information that will be resourceful to them. As the children grow, they require the same information that is provided to them for placement, diagnosis, etc. Then they would have understood the type, degree, and audiogram of their hearing loss in order to respond to inquiries about it and comprehend what it entails. They must comprehend how language, education, and literacy are affected by hearing loss. They must be able to speak up for themselves, ask for assistance when necessary, and articulate the issues they are facing.

Considering the necessity and benefits of family health rooted in REBT in past studies,^[21,25,75]^ as well as current research, this study suggests the establishment of a national institute to oversee the implementation of the REFHM principles and techniques, particularly in Sub-Saharan regions. Within this institute, practitioners will focus on addressing the psycho-emotional distress faced by parents of children with disabilities by providing rehabilitation for both the parents and the children. This psycho-educational and therapeutic program aims to help both parties adapt better to society. Establishing this institute in various locations will help achieve the intervention’s goals on a larger scale.

### 4.2. Conclusion

From the literature reviewed, we noted that there exist limited studies addressing emotional distress among parents parenting populations with HI in Enugu state, Nigeria. Some factors associated to emotional distress among others are the increase in blood pressure, sleepless nights, low self-esteem, and low self-confidence in managing a child with a disability; to this effect, the current study noted the need for more appropriate intervention like ours to support the parents to improve better. With the findings of this empirical study (REFHM), the existing knowledge gap seems to have been filled.

## Acknowledgments

We sincerely thank our participants and research assistants who showed commitment and dedication.

## Author contributions

**Conceptualization:** Anthonia Aneke, Chikodi Joy Anyanwu, Amanda Ugonna Ugwoezuonu, Moses Onyemaechi Ede, Ifeanyichukwu D. Okoro, Obiagali Loretta Aniaku, Ifeanyichukwu B. Agbigwe.

**Data curation:** Chikodi Joy Anyanwu, Amanda Ugonna Ugwoezuonu, Moses Onyemaechi Ede, Ifeanyichukwu D. Okoro, Obiagali Loretta Aniaku, Ifeanyichukwu B. Agbigwe.

**Formal analysis:** Chikodi Joy Anyanwu, Amanda Ugonna Ugwoezuonu, Moses Onyemaechi Ede, Ifeanyichukwu D. Okoro, Obiagali Loretta Aniaku, Ifeanyichukwu B. Agbigwe.

**Funding acquisition:** Anthonia Aneke, Chikodi Joy Anyanwu, Amanda Ugonna Ugwoezuonu, Moses Onyemaechi Ede, Beth Nnenne Oluka, Ifeanyichukwu D. Okoro, Nkechi T. Egenti, Obiagali Loretta Aniaku, Ifeanyichukwu B. Agbigwe, Joy Obiageli Oneli.

**Investigation:** Anthonia Aneke, Chikodi Joy Anyanwu, Amanda Ugonna Ugwoezuonu, Moses Onyemaechi Ede, Beth Nnenne Oluka, Ifeanyichukwu D. Okoro, Nkechi T. Egenti, Obiagali Loretta Aniaku, Ifeanyichukwu B. Agbigwe.

**Methodology:** Anthonia Aneke, Chikodi Joy Anyanwu, Amanda Ugonna Ugwoezuonu, Moses Onyemaechi Ede, Beth Nnenne Oluka, Ifeanyichukwu D. Okoro, Obiagali Loretta Aniaku, Ifeanyichukwu B. Agbigwe.

**Project administration:** Anthonia Aneke, Chikodi Joy Anyanwu, Amanda Ugonna Ugwoezuonu, Moses Onyemaechi Ede, Beth Nnenne Oluka, Ifeanyichukwu D. Okoro, Nkechi T. Egenti, Obiagali Loretta Aniaku, Ifeanyichukwu B. Agbigwe.

**Resources:** Chikodi Joy Anyanwu, Amanda Ugonna Ugwoezuonu, Moses Onyemaechi Ede, Beth Nnenne Oluka, Ifeanyichukwu D. Okoro, Obiagali Loretta Aniaku, Ifeanyichukwu B. Agbigwe, Joy Obiageli Oneli.

**Software:** Chikodi Joy Anyanwu, Amanda Ugonna Ugwoezuonu, Moses Onyemaechi Ede, Beth Nnenne Oluka, Ifeanyichukwu D. Okoro, Obiagali Loretta Aniaku, Ifeanyichukwu B. Agbigwe, Joy Obiageli Oneli.

**Supervision:** Anthonia Aneke, Chikodi Joy Anyanwu, Amanda Ugonna Ugwoezuonu, Moses Onyemaechi Ede, Beth Nnenne Oluka, Ifeanyichukwu D. Okoro, Nkechi T. Egenti, Obiagali Loretta Aniaku, Ifeanyichukwu B. Agbigwe, Joy Obiageli Oneli.

**Validation:** Anthonia Aneke, Chikodi Joy Anyanwu, Amanda Ugonna Ugwoezuonu, Moses Onyemaechi Ede, Beth Nnenne Oluka, Ifeanyichukwu D. Okoro, Nkechi T. Egenti, Obiagali Loretta Aniaku, Ifeanyichukwu B. Agbigwe, Joy Obiageli Oneli.

**Visualization:** Anthonia Aneke, Chikodi Joy Anyanwu, Amanda Ugonna Ugwoezuonu, Moses Onyemaechi Ede, Beth Nnenne Oluka, Ifeanyichukwu D. Okoro, Nkechi T. Egenti, Obiagali Loretta Aniaku, Ifeanyichukwu B. Agbigwe, Joy Obiageli Oneli.

**Writing – original draft:** Anthonia Aneke, Chikodi Joy Anyanwu, Amanda Ugonna Ugwoezuonu, Moses Onyemaechi Ede, Beth Nnenne Oluka, Ifeanyichukwu D. Okoro, Obiagali Loretta Aniaku, Ifeanyichukwu B. Agbigwe.

**Writing – review & editing:** Anthonia Aneke, Chikodi Joy Anyanwu, Amanda Ugonna Ugwoezuonu, Moses Onyemaechi Ede, Beth Nnenne Oluka, Ifeanyichukwu D. Okoro, Nkechi T. Egenti, Obiagali Loretta Aniaku, Joy Obiageli Oneli.
